# Appraising California Zinfandel Exposure to Wildfire Smoke Using Natural Product Phenolic Diglycoside Biomarkers

**DOI:** 10.1021/acs.jafc.2c04807

**Published:** 2022-09-08

**Authors:** Phillip Crews, Paul Dorenbach, Gabriella Amberchan

**Affiliations:** † Department of Chemistry and Biochemistry, University of California, Santa Cruz, California 95064, United States; ‡ SC Laboratories Inc, Santa Cruz, California 95060, United States

**Keywords:** smoke taint, phenols, diglycosides, zinfandel grapes, zinfandel wines, wildfire smoke exposure, MS/MS multiple ion reaction monitoring, winemaking, sensory evaluations

## Abstract

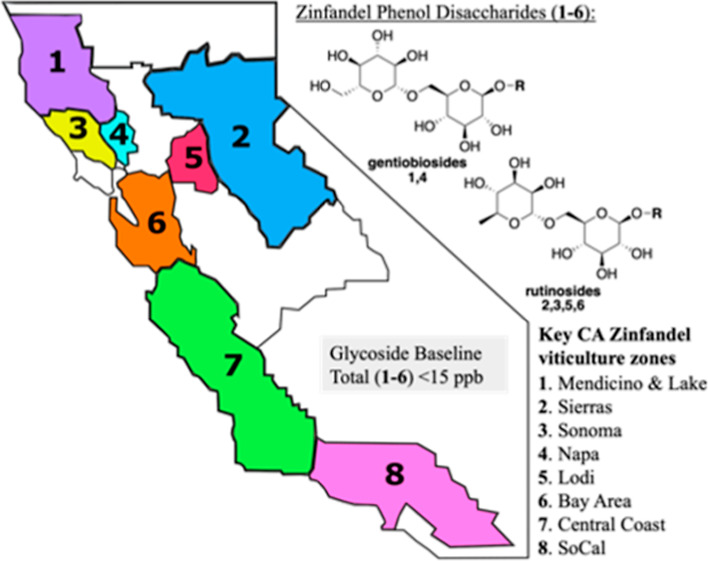

Zinfandel grapes are ubiquitous in California and its wine quality could be negatively impacted from wildfire smoke. Thus, the occurrence of fires prior to grape harvest presents a persistent problem to both viticulture and enology processes. This is the first broad study on Zinfandel to investigate wine quality defects produced by natural wildfires. The project, guided by UHPLC separations and MS^2^ multiple reaction monitoring, involved measuring natural product phenolic diglycosides (PDs) bioaccumulated in grapes, and expands outcomes published in 2022 by our team (called the Santa Cruz Campaign, SCC). The plan was implemented by exploiting a panel of six marker PDs **1**–**6** and their deuterated analogues. Examined in the study were 24 different Zinfandel wines obtained from 2016 to 2021 vintages of nine different American Viticulture Areas (AVAs) that were also within five of the eight California Zinfandel viticulture zones. The goal was to extend understanding on PD variations using patterns that possibly change as a function of appellation and fire intensity. Preliminary data was obtained to examine the relative amounts of PDs localized in berry skin versus pulp. The baseline of <15 ppb was proposed by surveying 18 distinct unsmoked Zinfandel wines. It was proposed to estimate the smoke impact on other Zinfandel wines by using seven PD ppb concentrations categories. A pilot study was also launched to assess conclusions by comparing ppb-based ratings versus sensory evaluation quality estimates. General findings presented herein should provide an important foundation to build understanding of using PD patterns to forecast possible Zinfandel wine wildfire damage.

## Introduction

Destructive wildfires continue annually in California wine country and appear to be catalyzed by droughts, heat waves, decades of overgrown forests, and population increases. It is now clear that wildfire smoke can undermine the quest to transform premium grapes into high quality wines, because of “smoke taint.”^1−3^ Recent comprehensive reviews^4,5^ summarized understanding, accumulated up to 2015, of a unique biochemical-driven phenomenon involving the capture of volatile phenols by grapes when exposed to wildfire smoke. A significant negative outcome of undesirable flavors often arises in the final wines made from smoke exposed grapes. These are often described as having bitter, smoky, astringent, and/or ashy aftertastes. Underscoring the magnitude of this problem, which can derail fine winegrowing, is that 13 of California’s 20 most destructive wildfires have occurred between 2016 and 2021 (see Tables, Supporting Information), and in 2020 about $600M of wine grapes in California were not picked because of concerns that mild to excessive smoke exposure could result in massive damage to wine quality.^6^

Not unexpectedly, much space in the recent news focuses on Cabernet Sauvignon to describe the problem of wine smoke taint—as illustrated by the partial title “protect California’s prized Napa Cab from the aftertaste of wildfires.”^7^ By contrast, Zinfandel has ostensibly escaped the rigorous attention given to Cabernet Sauvignon. This is surprising because of its prominence and long history in California. Introduced during the Gold Rush in the 1850’s, Zinfandel was the number two red grape crushed in California during 2021 (291,524 tons) versus Cabernet Sauvignon (590,249 tons).^8^ The project described herein was partially motivated because: (a) in the 1990’s the Zinfandel Heritage project was founded to preserve its living genetic resources by curating, in the UC Davis Foundation Plant Services program, grapes planted before 1930, (b) at one point in history (Aug 2006), Zinfandel was proposed by the California assembly to be the official state grape, (c) recent records show that in 2019 over 39,500 acres of Zinfandel are in California, and (d) in 2022 about 44 of the 58 counties in California grow Zinfandel.

**Scheme 1:**
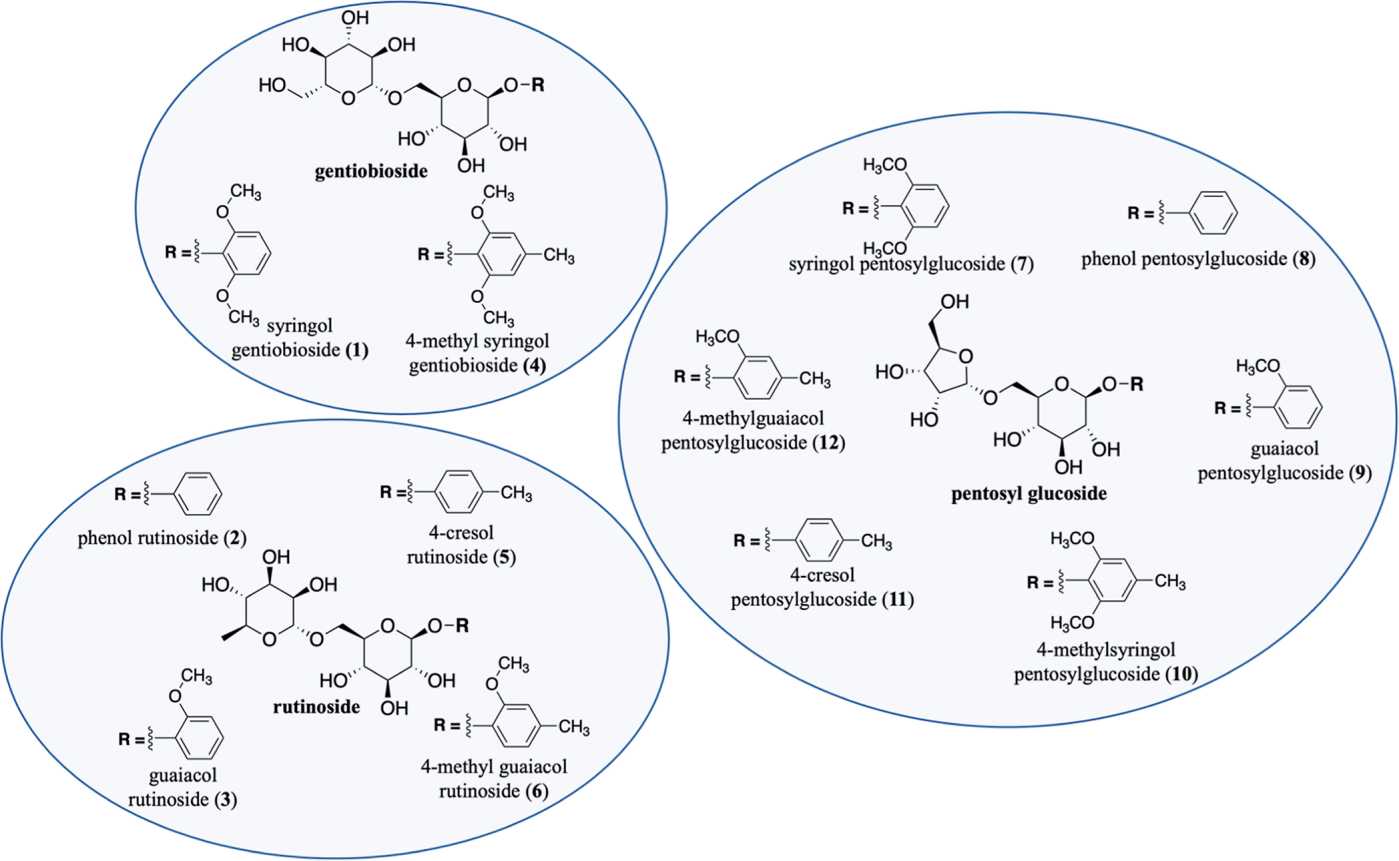
Three Major Classes of Phenolic Diglycosides (PDs) Used as Smoke Taint Wine Biomarkers and Their Specific Analogues, **1–12**

Widely recognized is that evaluating grapes or wines for their content of volatile phenols or phenolic glycosides, created during wildfires, provide useful insights to guide winemaking operations.^9^ Early on, in 2003, assessing the risk of smoke taint in grapes involved quantitation of just two volatile phenols, guaiacol and 4-methylguaiacol to reliably identify a smoke-affected wine.^10^ Eventually, attention turned to phenolic glycosides as better indicators to assess the potential negative impact of smoke taint in grapes and wines.^4,11^ It was also recognized that the concentration of free volatile phenols in smoke impacted grapes is very low and almost identical to the background level of that found in unsmoked grapes.^5,12,13^ Currently, fundamentals gathered from decades of research in Australia has defined useful workflows that use two metabolite types as biomarkers to estimate quality assessments. The compounds of the first group possess uncomplicated molecular structures and also have unpleasant organoleptic characteristics; consequently, they are rather easy to detect. This family currently consists of the two phenol containing molecules mentioned above plus another eight (three cresols, syringol, 4-methylsyringol, phenol, eugenol, and 4-ethylguaiacol). Constituents of the second metabolite group present a challenge to detect because they have rather intricate molecular structures alongside their benign sensory properties. The latter class currently entails 3 major families divided into 12 distinct phenol disaccharides (Scheme 1).^9,11^

The different bioanalytical approaches to detect the metabolite types, especially those shown in Scheme 1, have been recently discussed.^9^ Overall they include systems that: (a) directly measure free volatile phenols using GC–MS, or (b) that indirectly estimate total volatile phenols by sample hydrolysis (acid or enzymatic) followed by GC–MS analysis, or (c) that directly measure bound glycosylated volatile phenol compounds [phenolic diglycosides (PDs)] using mass spectrometer multiple reaction monitoring (MRM) data. The results from the first two methods are sometimes equivocal^9,11^ and outcomes can be difficult to interpret. As noted above, the concentration levels of free volatile phenols are often very low (<10 ppb), and data obtained for total volatile phenols involve indirect determinations with the potential for compound decomposition during the hydrolysis step. A game changer involved switching to the measurement of the phenolic glycoside (PG) molecules, typically accumulated in smoke impacted grapes and their wines at elevated concentrations (i.e., >100 ppb or more). These PGs (Scheme 1) can be found primarily as phenolic diglycosides (gentiobiosides, rutinosides, and pentosylglucosides). Alternatively, other minor analogues can be present as phenolic monoglycosides^14^ or phenolic triglycosides;^15^ however, it is the phenolic diglycosides that are the most stable and persistent at harvest and throughout wine aging.^9,15^ As a more recent development, an elegant study involving synthesis coupled with MS/MS CID high mass accuracy fragmentation analysis has identified additional guaiacol glycosides, including phenolic glucosyl glucosides, phenolic pentosyl glucosides, and rutinosides in the evaluation of smoke impacted berries grown in Canada.^16^ Finally, the list of potential phenolic glycoside markers has been greatly expanded based on an AI approach using MS/MS fragmentation patterns to putatively characterize an array of phenolic glycosides present in a 2017 modestly smoke impacted Napa Valley Cabernet Sauvignon wine.^15^ Curiously, while that study identified 31 volatile phenolic glycosides, none of the compounds shown were identical to those summarized in Scheme 1. In summary, our team recently concluded^9^ that the best approach incorporates the direct measurement of six bound glycosylated phenols (**1–6**).

**Figure 1:**
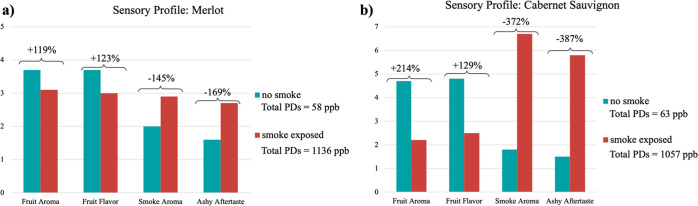
Comparison of wine published data of composition and sensory rating for non-smoke exposed and exposed to artificial smoke (1 h). (a) Merlot wine data for non-smoked samples (total PDs = 58 ppb) vs smoked samples (total PDs = 1136 ppb) and the sensory rating differences of: fruit +119% or +123%, vs smoke character of −145% or −169% based on a scale from 1 to 5.^14^ (b) Cabernet Sauvignon wine data for non-smoked samples (total PDs = 63 ppb) vs smoked samples (total PDs = 1057 ppb) and the sensory rating differences of: fruit +214% or +129%, vs smoke character of −372% or −387% based on a scale from 1 to 7.

Overall, there is wide interest among professionals engaged in viticulture and enology to quantitively and rapidly forecast the negative impact of wildfires on grapes and wines. Presented below is the first comprehensive study to quantitate smoke faults in Zinfandel grapes exposed to natural wildfires and the findings disclosed herein extend the power of metabolomics-driven data to evaluate Zinfandel, similar to that found to be valuable for California Cabernet Sauvignon. However, a confounding issue was identified while seeking to calibrate lessons learned from past studies. Essentially no data published to date merges appraisals of: (a) quantitation of the extent to which smoke containing volatile phenolics, created from natural wildfires, permeate a vineyard, (b) berry and/or wine bioanalytical data especially phenolic glycoside levels compiled for multiple wines of the same varietal, and (c) sensory properties for collections of smoke impacted wines annotated with their levels of phenolic glycosides. In addition, other than the small assemblage of results on Zinfandel from our prior publication,^9^ no other reports exist for Zinfandel. In this context, the closest relevant results are those recently published (Figure 1) for an Australian Merlot^14^ and an Australian Cabernet Sauvignon,^17^ whose grapevines were exposed after veraison to artificial smoke (1 h). Highlights of the trends represented in these excellent works are as follows: (a) there is a large spread in the ppb sums (>994 or >1078) for the PDs for the non-smoke exposed versus smoke exposed wines, and large ppb values of the latter indicate the grapevines were exposed to intense smoke. (b) Very small, but distinct numerical sensory score differences are also evident in the four sensory profiles tabulated in Figure 1 for fruit and smoke character ranging from +123%/+214%, or −169%/–387%. (c) The preceding trends imply that merger of bioanalytical and sensory evaluation data could greatly inform quality evaluations in the Zinfandel project, especially for heavily smoke impacted samples, as estimated by PD quantitation.

The lack of knowledge published about what happens to Zinfandel grape phenolics due to wildfire smoke exposure is striking. Thus, the goal of this project was to obtain broad understanding about Zinfandel and extend insights learned during the study by the Santa Cruz Campaign (SCC) of Cabernet Sauvignon and other red grapes.^9^ A first goal in the experimental design was to gather samples from the key California Zinfandel viticulture zones. Thus, the first phase of the project involved the direct measurement of six bound glycosylated phenols (**1–6**) and our survey was conducted on 24 California Zinfandel wines from nine distinct American Viticulture Areas (AVAs) across the 2016–2021 vintage years. A concurrent goal was to establish phenolic diglycoside baseline data for Zinfandel wine unimpacted by smoke that included sampling different vintages from varying AVAs. Our working hypotheses was that quantitating marker natural product PDs of normal versus smoke impacted grapes/wines would provide valuable input on estimating the extent of smoke taint impact on specific Zinfandel wines. The overarching goal was to evaluate a collection of wines subjected to varying levels of wildfire smoke to assess the accuracy of correlating PD sums in order to score varying levels of wine quality diminishment due to smoke taint. Another element of this project involved direct metabolomic analyses in order to avoid errors associated with workups using glycoside hydrolyses steps or inaccuracies associated with measurement of very low concentrations of free volatile phenols.^11^ The team also recognized that including a rigorous sensory evaluation component to the workflow was important, but this step required a comprehensive design,^1,14,17,18^ well beyond a metabolomics quantitation effort. Thus, it was concluded that for the initial study it was unrealistic to engage in a rigorous sensory evaluation; consequently, such a comprehensive effort was set aside for a second phase of the project to be conducted in due course. Alternatively, a small pilot study was conducted on 10 samples to compare ppb concentrations with sensory evaluation notes.

## Materials and Methods

### General Details

LC–MS grade acetonitrile (CH_3_CN), methanol (MeOH), isopropanol (IPA), and water (H_2_O) were purchased from the U.S. division of VWR International (Radnor, PA). The LC–MS grade ammonium formate and reagent grade sodium hydroxide (NaOH) were purchased from Sigma-Aldrich (St. Louis, MO). Each reference standard, **1**–**6** and their deuterated analogue, were purchased from Toronto Research Chemicals (TCW, Toronto, ON, Canada). All analytical runs were carried out at SC Laboratories, Santa Cruz, CA and used the same columns and analysis data collection methods as previously reported.^9^ Method validation protocols were followed from those previously described.^9^ The research described herein implemented analytic measurements that began with workups on SPE-treated samples subjected to UHPLC separations. These steps used Phenomenex Strata-X 33 μm polymeric reversed-phase SPE cartridges and Raptor ARC-18 100 × 4.6 mm, 2.7 μm particle HPLC columns purchased from Phenomenex (Torrance, CA) and Restek Pure Chromatography (Edmond, OK), respectively.

### Grape Juice Sample Preparation for Phenol Diglycoside Analyses

The Zinfandel grape sample (2 kg) coded El Dorado 21-G-2 (ED-2-2021) was hand harvested well after veraison on 9/27/2021 as clusters on-the-vine from distinct sites in the vineyard block. The fruit was significantly impacted by the Caldor fire smoke (8/14/21 to 10/21/21). The grapes were stored in Ziplock Polyethylene Gallon Freezer bags, refrigerated, and shipped (2 days) to UC Santa Cruz. They were additionally divided into two equal batches then kept frozen until a further workup. The initial PD analysis was carried out on the first batch in Nov 2021. The sample workup on batch two began on 5/1/2022 and commenced once the frozen grape clusters were allowed to thaw over about 3 days at 40 °F. This provided three separate juice samples (0.6 kg of the thawed destemmed berries). The workflow to obtain distinct juice samples for subsequent PD analyses involved: (a) *residual juice* (300 mL located at the bottom of the bag, moderately red color, and leaked from the whole berries during the thawing) ELDO-21-G-2, Liquid, total glycosides = 267.6 ppb, 14° Brix, pH 3.47; (b-1) *juice-pulp IA*, (from 275 g of berries) total glycosides = 238.0 ppb, 17° Brix, pH 3.53; (b-2) *juice-pulp IB*, (from 326 g of berries) ELDO-21-G-2, Juice Pulp, total glycosides = 245.2 ppb, 16.5° Brix, pH 3.51; (c-1) *skins IA* (127 g, extracted 5 days with 20% ethanol) ELDO-21-G-2, Skin, total glycosides = 110.3 ppb; and (c-2) *skins IB* (174 g, extracted 5 days with 20% ethanol) total glycosides = 97.2 ppb. The liquids from the juice-pulp samples (b-1 and b-2) or skins (c-1 and c-2) were obtained by pressing-filtration to capture the grape marc using a six-inch stainless steel mesh strainer.

### Berry or Wine Grape Juice Sample Preparation for UHPLC-MS/MS Analysis

The first steps in processing the berry grape juice and wine supernatants were analogous to that published by various labs^2,9,11,15,16^ and began with centrifugation followed by SPE cleanup or just the latter step depending upon turbidity. Typically, samples were centrifuged at rt 4000*g* in an Restek machine (model 755-24). Further cleanup via filtration was carried out using a Strata-X 33 μm Polymetric reversed-phase SPE cartridge that was conditioned with 2.0 mL of CH_3_CN followed by 2.0 mL of H_2_O. Then, 1.0 mL of the sample were loaded onto the SPE cartridge. The sample was washed with 1.0 mL 0.1 M aqueous NaOH followed by 2.0 mL of H_2_O. The sample was then eluted with 1.0 mL of 40% CH_3_CN in water. Next, as previously described,^9^ samples were processed using a LX-50 UHPLC coupled with a QSight 210 triple quadrupole mass spectrometer (Perkin Elmer, Waltham, MA). A sample (3.0 μL) was injected onto a Raptor ARC-18 with a Raptor ARC-18 guard column. The autosampler temperature was set to 10 °C and the column oven was set to 30 °C. Mobile phase A consisted of 1.0 mM ammonium formate in H_2_O with 0.1% formic acid, and mobile phase B consisted of 1.0 mM ammonium formate in MeOH with 0.1% formic acid, with a flow rate of 0.8 mL/min. The pump gradient started with a 0.5 min hold at 30% B, followed by a linear gradient from 30% B to 50% B over the course of 10 min, followed by a 100% B column flush for 5 min, and then returned to 30% B for 2 min to equilibrate.

### Natural History Notes for the Named Fires Impacting the Zinfandel Vineyards

**Figure 2:**
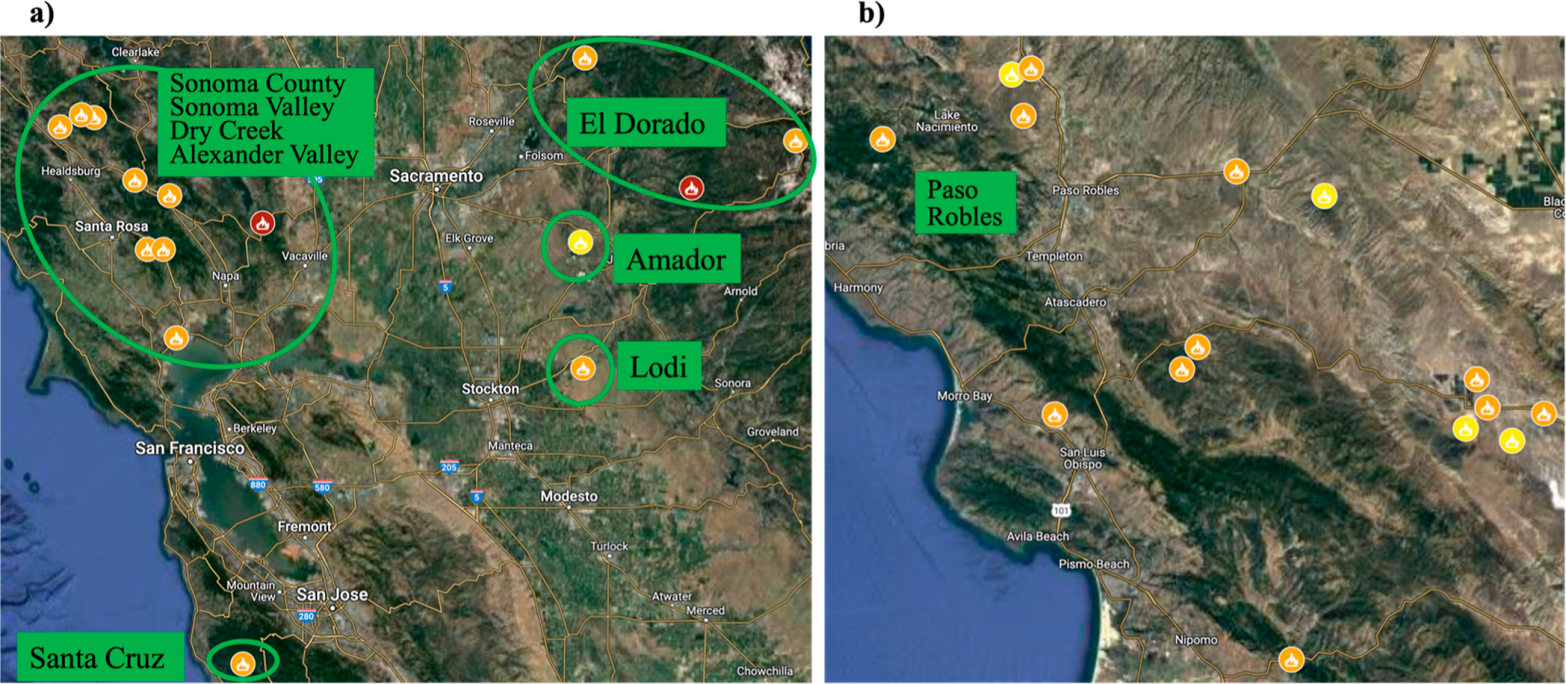
Named fires in CA Zinfandel viticulture zones (VZ) containing AVAs (green boxes) between 2016 and 2021. (a) In the AVAs of Amador (VZ-2), El Dorado (VZ-2), Santa Cruz (VZ-6), Sonoma County (VZ-3), Sonoma Valley (VZ-3), Alexander Valley (VZ-3) Dry Creek (VZ-3), and Lodi (VZ-5) there were 2 extreme fires (>100,000 acres, red), 12 major fires (1000–99,999 acres, orange), and 1 large fire (500–999 acres, yellow). For more information see Tables S2–4, S6, and S7. (b) In San Joaquin County, home of Paso Robles (VZ-7), 11 major fires (1000–99,999 acres, orange), and 4 large fires (500–999 acres, yellow) occurred. For more information see Table S5.

All the finished wines came from nine AVAs, including some that might be designated as containing “old-vines.” This assemblage was also from the 2016–2021 vintages from vineyards within five CA Zinfandel viticulture zones (table of contents graphic), and their potential proximity to the sites of 154 fires are shown in Figure 2 and in Tables S1–S6. The most smoke impacted vineyards were assumed to be from the sites of the two extreme fires (>100,000 acres) shown by the red icons in Figure 2A. Also displayed in Figure 2 are the positions of the 23 major fires (1000–99,999 acres as orange icons), and the locations of the 5 large fires (500–999 acres as yellow icons).

### Wine Samples for Phenol Diglycoside Analyses

Quantitative data are reported and discussed below for 23 California Zinfandel finished wines (60 mL samples) and similar data for one other sample can be found in a prior publication.^9^ The average percentage of PDs **1**–**6** in the 24 Zinfandel wine samples versus categories of smoke intensity are shown in Figure S1. All the wines were made from vineyards located in nine distinct AVAs, including some that could be designated as containing “old-vines.” This demonstration set also ranged from entries never exposed to wildfire smoke versus those that were heavily smoke impacted as discussed above. The tally of ppb concentrations for phenol diglycosides (PDs) **1**–**6** were measured on 18 samples as described below, and the previously published quantitation data (ppb)^9^ are also shown herein for the six wines coded by a * in data sets. A full summary of the wine sample codes according to their sources appear in Table 1. To add clarity, additional annotations for each of the samples include the % Zinfandel composition (Figure S1) along with a rough estimate of smoke exposure. An overview of the 23 wine samples by Viticulture Zones (VZs) is also as follows: (i) VZ#2, the Sierras, *n* = 4 (+1 not shown); (ii) VZ#3, Sonoma, *n* = 14; (iii) VZ#5, Lodi, *n* = 1; (iv) VZ#6, Bay Area, *n* = 3; and (v) VZ#7, Central Coast, *n* = 1.

**Table 1:**
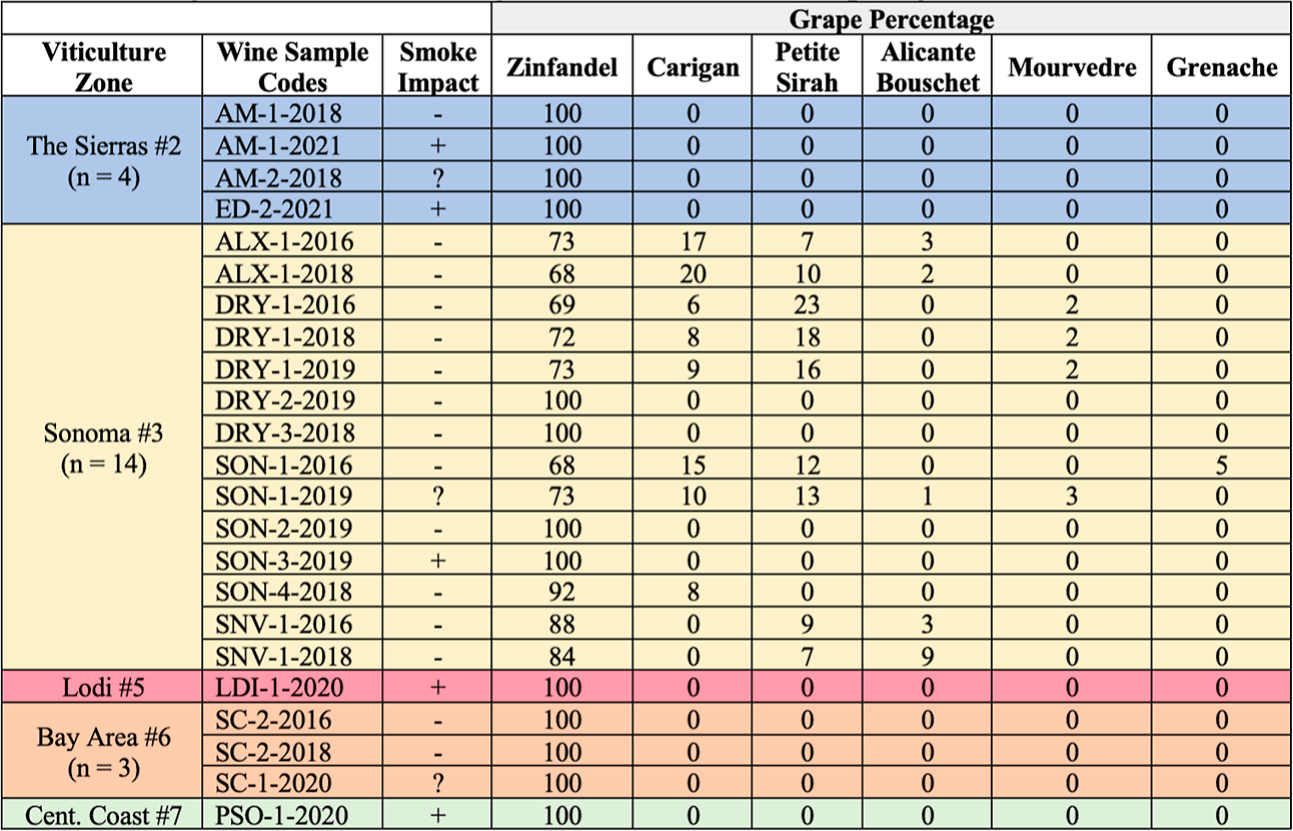
Summary of 23 Zinfandel Wine Samples Evaluated Across Five of Eight Key CA Viticulture Zones^a^

a See graphical abstract.

### Reference and Calibration Standards

Reference samples of each of the six phenol diglycoside compounds were used for internal standards to provide the quantitative data. These compounds are shown in Scheme 1 and also include the use of their isotopically labeled counterparts. Standards were purchased from Toronto Research Chemicals (TCW, Toronto, ON, Canada) and included: syringol gentiobioside (**1**), syringol gentiobioside-*d*_6_ (**1-*d***_**6**_), phenol rutinoside (**2**), phenol rutinoside-*d*_5_ (**2-*d***_**5**_), guaiacol rutinoside (**3**), guaiacol rutinoside-*d*_3_ (**3-*d***_**3**_), 4-methylsyringol gentiobioside (**4**), 4-methylsyringol gentiobioside-*d*_6_, (**4-*d***_**6**_), 4-cresol rutinoside (**5**), 4-cresol rutinoside-*d*_7_ (**5-*d***_**7**_), 4-methylguaiacol rutinoside (**6**), and 4-methylguaiacol rutinoside-*d*_3_ (**6-*d***_**3**_). Calibration standards were prepared by combining the six reference standards at concentrations ranging from 0.5 to 1000 ng/mL. Internal Standard (ISTD) solutions were prepared by combining the six isotopically labeled compounds such that the approximate on-instrument concentration was 250 ng/mL. The UHPLC conditions described above gave distinct retention times for all 6 standards and their deuteron analogs with the latter member of each pair always eluting first as published previously.^9^ These retention time patterns were also consistent with negative ion ESI-MS^2^ data used to visualize the diagnostic [M + HCOO]^−^ ions together with other key fragments [M–H], and M – [substructure (phenol or hexose)]^−^ ions analogous to those shown in past publications.^9,15,16^

LC–MS/MS data was processed using the Simplicity (v.1.8) software package (PerkinElmer, Waltham, MA). For MS^2^ analysis, phenolic diglycosides were detected in the MRM mode by negative-mode electrospray ionization (ESI). A source temperature of 310 °C was used, with an electrospray voltage of −4200 V, a nebulizer gas of 350 °C, and a drying gas of 120 °C. Parent ions formed from formic acid adduct ions [M + HCOO]^−^ to the most abundant fragments were used for quantifying and confirming the bound PD. Three mass transitions were used for each compound with the highest mass fragment used for quantification, as it is theoretically the most selective. The CID collision energies varied from 20 or 42 V and were optimized by the following standard instrument software’s autotune function. Listed next are the mass transitions and retention times (*t*_R_) for the six bound PD compounds (see, ref (9) Supporting Information for the full CID fragmentation network). The data set includes: syringol gentiobioside (**1**) (*m*/*z* 523.3/323.2, 523.2/119.1, 523.3/89.1, 2.49 min), phenol rutinoside (**2**) (*m*/*z* 447.2/307.2, 447.2/163.1, 447.2/103.2, 2.74 min), guaiacol rutinoside (**3**) (*m*/*z* 477.3/307.2, 477.3/163.2, 477.3/103.1, 3.21 min), 4-methylsyringol gentiobioside (**4**) (*m*/*z* 537.4/323.1, 537.4/119.1, 537.4/89.1, 3.75 min), 4-cresol rutinoside (**5**) (*m*/*z* 461.3/307.2, 461.3/163.2, 461.3/103.1, 4.36 min), and 4-methylguaiacol rutinoside (**6**) (*m*/*z* 491.3/307.2, 491.3/163.2, 491.3/103.1, 4.91 min).

### Highlights of Other Analyses

The workflow to validate analyte calibration curves was described previously^9^ and was employed herein. First, calibration curves were linear with *R*^2^ > 0.995 with a calibration range of 0.5 ng/mL to 1000 ng/m. Second, spiking reference standards at two concentration levels (50 and 500 ng/mL) involved two sample types (wine and fresh juice). Third, estimates of data quality were again conducted; recovery and accuracy were within 90–130%, and precision (calculated as percent relative standard deviation) less than 5%. Fourth, evaluation of precision percent was determined and estimating the limitation of detection–quantitation in ppb or μg/L for **1**–**6** used runs with the deuterium analogues. Fifth, the uncertainty in percent detection also involved runs with **1**–**6** deuteron analogues to give the outcomes summarized previously. Finally, estimates of and the recovery of specific analytes varied from 89 to 108%.

## Results and Discussion

### Current Landscape Pertaining to Baseline PD Concentrations in Nonsmoke Exposed Wines

A 2013 publication demonstrated that unsmoked Australian Cabernet Sauvignon, Shiraz, and Chardonnay grapes possess the biosynthetic apparatus to create and store PD biomarkers, such as **1**–**12**.^11,15,16,19,20^ However, today very little is known for Zinfandel regarding how these PDs can vary in concentrations in normal versus smoke impacted grapes from different viticulture areas. In fact, just one data set is currently available that proposed a preliminary baseline sum of **1**–**6** < 9 ppb based on data from just three California Zinfandel wines.^9^ Discussed below is the basis to confidently revise this value slightly upward. This outcome now aligns with information for non-smoke impacted Cabernet Sauvignon that is now mature. The SCC publication^9^ clearly showed the baseline sum of **1**–**6** < 6 ppb for California Cabernet Sauvignon (*n* = 21), which is very different to that <20 ppb recently revised downward in 2022 for Australia Cabernet Sauvignon.^2^ Currently, there is a somewhat perplexing situation pertaining to data packages used to document insurance claims to compensate for loss due to wildfire smoke. Fortunately, the U.S. Department of Agriculture’s Risk Management Agency (RMA) provides insurance to compensate for loss due to wildfire smoke, but physical damage must be documented using analytical baseline data showing elevated levels of free guaiacol and 4-methyguaiacol.^6^ The SCC group recommends that the lens be widened to incorporate baseline PD data which will be more diagnostic versus merely using the baseline of phenols to assess the extent that grapes have been exposed to damaging smoke.^9^

### Accumulation of PDs in 2021 Zinfandel Grapes and Wine from Heavy Smoke Exposure

Some steps in premium winemaking involve differential extraction of constituents from distinct grape berry regions. Relevant to this are the potential dissimilarities in the PD content of a ripe berry from: region A, skin (outer exocarp and exocarp); region B, pulp—flesh—mesocarp, and region C, pulp—flesh—endocarp plus seeds. Not well understood is exactly how the juice obtained from regions A–C differ in PD content among fractions described as: *free run* or the first light press from region B; *heavier press* or juice from region C; or *final press*—juice from region A. Currently, the most relevant insights about the distribution of phenolic glycosides (PGs) in grape sections are from an elegant 2011 study.^12^ That work investigated the distribution of PGs from lightly, artificially smoked (30 min) Australia Merlot grapes, by comparing their PG concentrations in grape homogenates versus those of “skins”, “pulp”, and “seeds.” Although data for specific molecular structures were not presented, “glucose–pentose” disaccharide concentrations (μg/berry) predominated in the homogenate and their abundance ratio for skins/pulp was ≅ 7.

**Figure 3:**
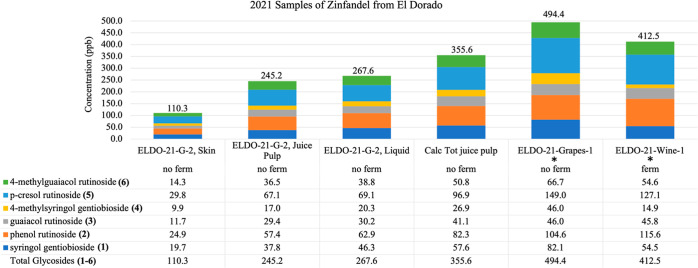
Quantitation of bound phenol disaccharides in 2021 Zinfandel grapes from the El Darodo (ELDO) AVA. This data examines the quantity of glycosides present in grape skins vs the juice when fermented or non-fermented. * Data from ref (9).

Another important perspective was provided by the properties measured for two intensely smoked 2021 Zinfandel grape lots from the El Dorado AVA, harvested on 9/27/2021 by the UCCE group.^9^ These samples consisted of those from Vineyard A = 2 kg and Vineyard B = 3 kg, which were kept frozen in duplicate Ziplock freezer bags until further workup. The bags #1 from both vineyards were immediately analyzed for the PD content and data for both have been previously published.^9^ For clarity, some results of Vineyard A are reiterated in Figure 3 (samples coded “ELDO-21-Grapes [or Wines]-1”). The totals of **1**–**6** in the grapes (or the final wines) were as follows:^9^ Vineyard A = 494.4 ppb (or 412.5 ppb) and Vineyard B = 654 ppb (or 821 ppb). Significantly, for both grape samples, the levels of all PD totals were very large, while the relative concentrations for **4** were quite small: Vineyard A, grapes\wines = 46\15 ppb; and Vineyard B = grapes\wines = 14\5 ppb, respectively. As noted, the concentrations of **4** from both vineyards diminished during fermentation and the concentrations of constituents **2**, **5**, and **6** also diminished during fermentation for Vineyard A (grapes\wines from 320 to 297 ppb). However, a different pattern was observed for the Vineyard B sample as the concentrations of constituents **2**, **5**, and **6** substantially increased during fermentation (from 496 to 661 ppb).

In order to gain further understanding of these preceding patterns, additional analyses were undertaken on pre-fermentation samples of bag #2 from Vineyard A that was processed on 5/1/2022 (Figure 3). Three separate juice samples were obtained from the ca. 0.6 kg of the thawed material and were coded “ELDO-21-G-2.” These new samples included: (a) *residual juice or the liquid collected at the bottom of the bag*, 300 mL, 14° Brix, pH 3.47, “ELDO-21-G-2, Liquid” **1**–**6** = 267.6 ppb; (b) *juice-pulp IB*, 16.5° Brix, pH 3.51 from the pressing of berries, 326 g, “ELDO-21-G-2 Juice Pulp”, total glycosides = 245.2 ppb; and (c) *skins IA*, 127 g, remaining from the grape pressings and subjected to a 5 day extraction with 20% ethanol, “ELDO-21-G-2 Skin”, total glycosides = 110.3 ppb. It should be underscored that this is a preliminary data set and it clearly shows that the biosynthesis of significant concentrations of each of the biomarkers can occur in both the skins or pulp prior to fermentation.

There is one more circumstance deserving of brief discussion. It is noteworthy that large concentrations of natural product PDs are shown to be created and stored in several different regions of grape berries, owing to intense smoke exposure, post-veraison. Significantly, these data also proves that the biosynthesis of large concentrations of five of the six biomarkers can occur in both the skins and pulp prior to fermentation and that these compounds are very stable to fermentation yeasts. In fact, inexplicably, the overall levels of PDs measured for the sample of Vineyard B were larger in the wine versus that in the grapes. The consistent observation that **4**, formed in small amounts, can be labile during fermentation does not overly impact the conclusion that significant smoke taint damage can occur for Zinfandel grapes, which can be correlated to the concentrations of PDs. Thus, as further discussed below, the potential for smoke taint in Zinfandel can be easily forecasted prior by measuring the PD concentrations in macerated grapes, without carrying out a small batch-scale fermentation, often done by some professionals, anytime between veraison and harvest.

### Survey of 24 California Zinfandel Wines through the Lens of Nine AVAs and Different Vintages Based on Measuring Their Bound PDs

For 2020 in California, the combined Cal Fire US Forest service estimate of wildfires is staggering—8,649 fires and 4,304,379 acres burned. Alongside these facts, 76% of the counties in California have Zinfandel vineyards under cultivation; consequently, the continuing annual potential threat to these grapes from smoke taint is substantial. In contrast, no comprehensive studies have yet to be completed on Zinfandel to assess the value in using either free phenols or PDs (aka bound phenols) to evaluate the impact of wildfires on its wines. The present work sought to extend a preliminary effort represented in the SCC 2022 publication,^9^ reporting bound PDs of seven different Zinfandel wines from three Northern California AVAs: Sonoma County, El Dorado County, and Amador County. The previously published quantitation data^9^ for six/seven wines are shown in Figure 3 coded with a * and include a tally of ppb concentrations for PDs **1**–**6**. Importantly, the SCC team recognized a vital limitation—this previous work which included just three non-smoke exposed samples from only two AVAs. In contrast, Zinfandel vineyards are found in more than eight key California Zinfandel Viticulture Zones (VZs): (1) Mendocino and Lake Counties, (2) The Sierras Foothills, (3) Sonoma County, (4) Napa Valley, (5) Lodi, (6) The SF Bay Area, (7) Central Coast, and (8) Southern California. Logically, some important next steps in this research aimed to broaden the sample acquisition crusade and obtain enough data to establish a baseline concentration for non-smoke exposed Zinfandel grapes that span the entirety of these VZs.

**Figure 4:**
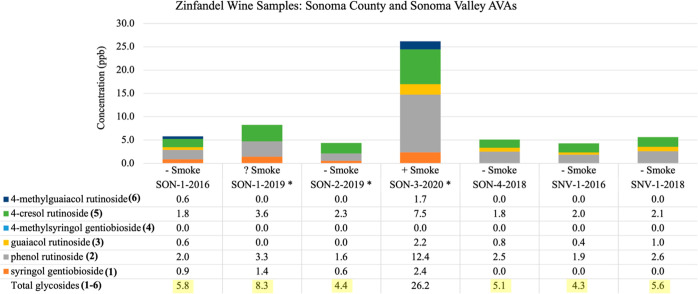
Quantitation of bound phenol disaccharides in Zinfandel wine samples across two California AVAs: Sonoma County (SON) and Sonoma Valley (SNV). Smoke codes ++ (heavy smoke), + (moderate smoke), – (no smoke), and ? (light to no smoke). * Data from ref (9).

**Figure 5:**
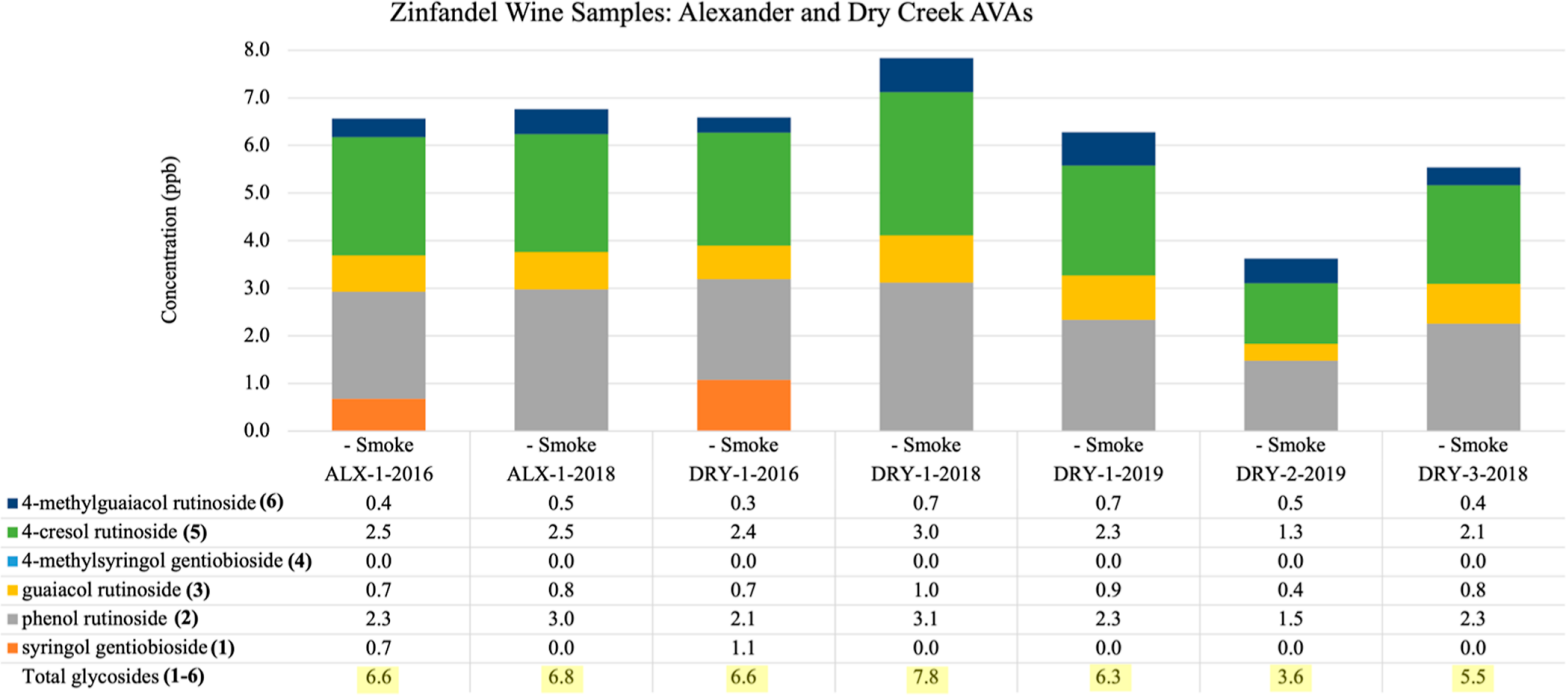
Quantitation of bound phenol disaccharides in Zinfandel wine samples across two California AVAs: Alexander (ALX) and Dry Creek (DRY). Smoke codes ++ (heavy smoke), + (moderate smoke), – (no smoke), and ? (light to no smoke). * Data from ref (9).

**Figure 6:**
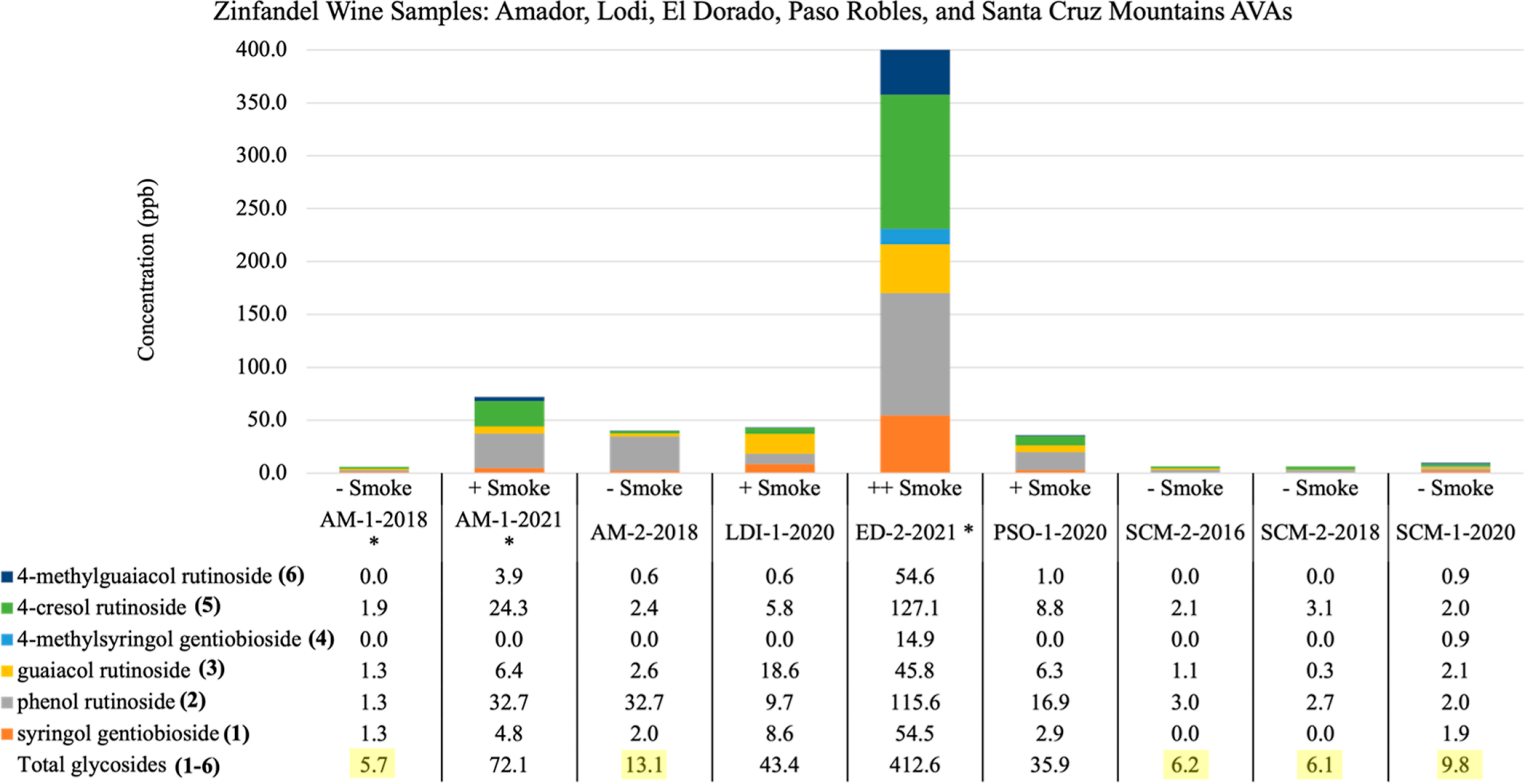
Quantitation of bound phenol disaccharides in Zinfandel wine samples across five California AVAs: Amador (AM), Lodi (LDI), El Dorado (ED), Paso Robles (PSO), and Sonoma Cruz Mountain (SCM). Smoke codes ++ (heavy smoke), + (moderate smoke), and – (no smoke).

The quest to accumulate several Zinfandel wines was successful and generated a portfolio of 17 additional Zinfandel lots from vintages 2016 and 2018–2020. Even though all of these included bottled wines from previous barrel aging, it should be underscored that the presence of marker compounds **1**–**6** can only arise from biosynthetic processes occurring in the vineyard and not during storage in (toasted) barrels.^9^ The contents of Table 1, Figures 4–6, and Figure S2 summarize key details for each wine including: the individual concentrations (ppb) of **1**–**6**, the total glycosides sums, the percent Zinfandel compositions alongside that of other grapes sometimes present (Carignan, Petite Sirah, Alicante Bouschet, Mourvedre, and Grenache), qualitative estimates of the smoke levels (“++”,“+”,“–”), the wine vintage, and the AVA. An additional matrix of information in Figure 2 displays: (a) the location of samples obtained from the nine AVAs, (b) the approximate boundaries of five/eight Zinfandel VZs in relation to the named fires between 2016 and 2021, and (c) information that can be cross correlated with each sample by scrutinizing sample codes in the experimental against the VZ information above in Table 1.

The data that follows next provides the foundation to review the findings of this study that are effective in predicting smoke impacts to Zinfandel wine quality damage from wildfire smoke. The scope of samples provides a nice perspective because they were drawn from five of the eight major Zinfandel CA VZs. Additional quantitative information is collected in Supporting Information, Tables S1–S7, and Figures S1–S2. An overview of the 24 wine lots from nine distinct AVAs are as follows. (A) There are 14 samples from VZ-3 (Sonoma) summarized in Figure 4, covering the Sonoma County and Sonoma Valley AVAs (*n* = 7); and also summarized in Figure 5 covering the Alexander Valley and Dry Creek Valley AVAs (*n* = 7). (B) Ten samples were from five AVAs including Amador, Lodi, El Dorado, Paso Robles and Santa Cruz Mountains and nine are summarized in Figure 6. Importantly, the expanded data assemblage establishes a total of**1**–**6**glycosides < 15 ppb as the amended baseline for unsmoked Zinfandel wines, providing a hypothesis to clearly define the upper level of PD concentrations for clean wines.

There were 18 samples in the Zinfandel set comprising the baseline wines, and this element of the experimental design is especially robust. These baseline samples are denoted with the yellow highlights and appear across all three of the AVA compilations (Figures 4–6). Among the baseline samples 13 are from VZ-3 (Sonoma) and the other five were distributed as follows: two from VZ-2 (Sierras) and three from VZ-6 (The SF Bay Area). The smoke taint impact on the remaining six samples was observed to be elevated relative to the baseline based on PD concentrations. The lots were ranked using the six categories previously introduced to describe the categories of smoke infestation for Cabernet Sauvignon by our SCC group and are now slightly revised for Zinfandel as shown in Table 2–top panel. A discussion on the additional preliminary steps taken in this study to justify these groupings is important, but for clarity will be outlined in a separate section that follows below. Thus, the remaining samples are divided into the following categories of smoke: one from VZ-3 (Sonoma) was light (15–30 ppb); three from VZ-2 (Sierras), VZ-5 (Lodi), and VZ-7 (Central Coast) were modest (31–100 ppb); and two from VZ-2 (Sierras) were severe (>399 ppb).

**Table 2:**
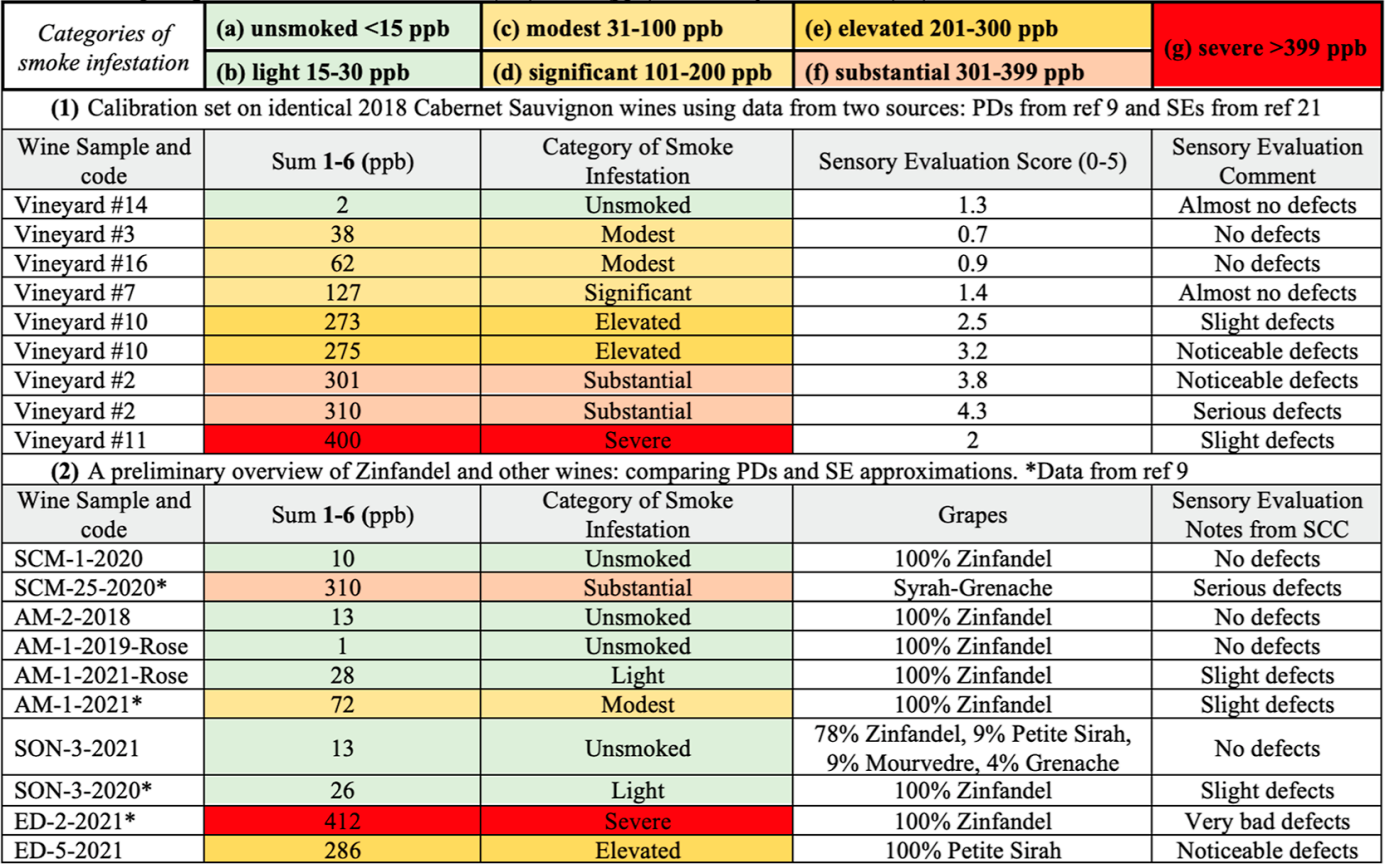
Comparing Bound Phenol Disaccharide (PD) Sums (ppb) to Sensory Evaluation (SE) Assessments

As noted above, the analysis of the Zinfandel PD concentrations indicated that compound **4** is inconsequential in the assessment of smoke exposure from both grapes and wine. Interestingly, regardless of smoke impact, compounds **2** and **5** were the most prominent PDs present. For instance, in the 18 non-smoked samples **2** and **5** were present in 38% and 35% of the total PDs, respectively (Figure S1). The light and moderate smoke impacted wines follow a similar pattern, albeit from a smaller sample set. In the two heaviest smoked samples, the percentage of **2** and **5** were almost equal (27 and 38%, respectively). Additionally, compound **6** doubles in quantity from the non-smoke (5%) to the heavy smoke (11%) but it remains drastically lower than the two major PDs. Additional samples at these three smoke-impacted levels would benefit further analysis of such patterns. Even so, these trends allude to the uniformity in which PDs appear across the various AVAs of CA Zinfandel wines. These results further highlight the importance of expanding the panel to include other biomarkers.

### Defining the Categories of Smoke Infestation for California Zinfandel Wines

Analysis of the varying Zinfandel PD concentrations summarized in Figures 4–6 can be facilitated by applying the categories of smoke infestation created by our team for Cabernet Sauvignon wines.^9^ For example, analogous to the outcome of the Cabernet Sauvignon study, the PD totals for **1**–**6** were used to define the upper level of concentrations for clean wines and six additional quality categories for higher ppb sums were proposed for smoke exposed lots. Thus, the PD data discussed below provides an important foundation to use PD patterns to forecast Zinfandel quality.

Now summarized in Table 2 is a rubric to estimate quality damage to Zinfandel that builds on outcomes from two studies of 14 distinct 2018 Cabernet Sauvignon wines (Napa and Lake AVAs).^11,21^ These Cabernet Sauvignon wines were differentially exposed to smoke from three major fires, causing a range in **1**–**6** concentrations from 2 ppb (no smoke) to 400 ppb (severe smoke). As shown next, it was encouraging to discover that for these Cabernet Sauvignon wines a general correlation can be established between the PD sums reported in 2022^11^ versus sensory evaluation (SE) assessments^21^ disclosed in 2020. First, the entries in Table 2 (part 1) with low ppb (2, 38, or 62 ppb) relate with the best SE scores (1.3, 0.7, or 0.9), and the best SE comments (“almost no defects” or “no defects”). Second, the selected entries with intermediate or high ppb (127, 273, or 310 ppb) match those with the intermediate or high SE scores (1.4, 3.2, or 4.3), but with varying SE comments (“almost no defects”, “noticeable defects”, or “serious defects”). Third, there are some disturbing outliers to the correlations of ppb values versus SE scores. For instance, Vineyard #11 must be categorized as severe based on PD analysis (400 ppb) and yet its SE score (2) is more akin to the descriptor of a modest to elevated PD ppb categorization. Even duplicate samples show some irregularities in the SE assessments (see Vineyard #10—two entries, or Vineyard #2—two entries), while the PD analysis based on ppb remains relatively consistent. Consequently, there seems to be an important caveat—measuring bound PDs can be accurately done; whereas SE assessments, even by trained panels, are sometimes imprecise.

Regarding another important point, a non-rigorous approach was taken during this study to initially correlate conclusions drawn from the Zinfandel seven-level ppb rating versus those from sensory evaluation estimates. A set of ppb values are collected in Table 2 (part 2) for seven 100% Zinfandel samples, one Zinfandel blend, and for two other grape types sometimes used in Zinfandel blends. Also tabulated for each wine is a preliminary sensory quality estimation provided by the SCC team and winemaker volunteers (*n* = 3–7). A palpable correlation was observed between the ppb sums versus the sensory evaluation notes. Also, six out of the seven smoke infestation categories were represented in this small collection of 10 wines. Correspondingly, these preliminary trends appear to be useful, but in the future additional validation will be needed by including three or more samples from each category of smoke infestation in a study that involves parallel biomarker PD measurement and rigorous sensory evaluation by >10 trained judges.

Correspondingly, it is important to note that while this study did not include documented “old-vine” Zinfandel fruit (i.e., grapes planted before the 1930’s), it is assumed that the patterns discussed above would be relevant to identify unsmoked fruit for such heritage plants. We also propose that the concentration boundaries discussed above can be applied broadly to search for non-smoke impacted Zinfandel grapes grown in all VZs of California. Finally, it is also tempting to assume that PD patterns reported herein can be extrapolated to evaluate closely related Zinfandel clones including Primitivo, which is somewhat common in California. Or in the future, to Pribidrag vines (from Croatia), which are closely related to Zinfandel, and have been recently planted (2015) in the Dry Creek Valley and in the Santa Cruz Mountains AVAs.

In summary, this work reports some of the first quantitative measurements of PDs bioaccumulated in premium California Zinfandel grapes and wines due to wildfire smoke. Established analytical professionals, including those at the AWRI^2^ or ETS,^22^ remain silent on the background concentrations of smoke marker metabolites in Zinfandel from either grapes or wines. The results reported above were assembled to contribute new information by successfully merging strategies of bioanalytics, oenology, and focused collections of grapes and wines from vineyards exposed to varying smoke (see Figure 2) to estimate the impact of wildfires on wine quality. Metabolomic analyses were guided by UHPLC separations and MS^2^ multiple reaction monitoring and were greatly facilitated by exploiting a classic panel of six marker PDs and their deuterated analogues (**1**–**6**). In the future, these outcomes will help to guide wine fault analyses based on merging exact concentrations determined for PDs with ratings from qualitative sensory evaluation trials on finished wines.

A total of 24 distinct Zinfandel wine samples were examined in this and in a previously published preliminary study^9^ from 2016 to 2021 vintages from nine AVAs. The additional new data reported here establishes total glycosides <15 ppb as the baseline data for unsmoked Zinfandel wines. Overall findings presented provide an important foundation of using PD patterns to forecast Zinfandel quality defects resulting from wildfire events. Re-examination of two different datasets^9,21^ from California Cabernet Sauvignon wines substantiated the proposal to divide these into seven quality categories. Similarly, the smoke impact on Zinfandel wines can also be divided into these same seven categories. In fact, a successful pilot study was undertaken to begin the process of validating quality conclusions drawn for Zinfandel by comparing the seven-level ppb ratings versus estimates from qualitative sensory evaluations.

In the future, using quantitative PD data along with outcomes from sensory evaluations represent the pathway to design mitigation treatment of smoke impacted Zinfandel grapes or wines possibly via emerging, minimally invasive strategies headed by ozone treatment rather than the somewhat intrusive reverse-osmosis or solid phase adsorption.^14^ Finally, additional metabolomic evaluations are needed to redesign the current portfolio of phenolic disaccharides used as biomarkers for Zinfandel and possibly other varietals. It is clear, that among the Zinfandel biomarkers **1**–**6**, the least abundant and most labile analogue **4** should be replaced. Future candidates for the biomarker panel could include diglycosides containing pentosyl moieties, such as those shown in Scheme 1. Other candidates could include phenolic biomolecules possessing carbohydrate structures related to those shown in Scheme 1 but with phenolic residues having a different pattern of heteroatom constituents.^23^

## Supporting Information Available

Total number of fires between 2016 and 2021 in the five selected CA Zinfandel viticulture zones (and the 9 specific AVAs); 2016–2021 Fire map of AVA: Amador; 2016–2021 Fire map of AVA: El Dorado; 2016–2021 Fire map of AVA: Lodi; 2016–2021 Fire map of AVA: Paso Robles. ; 2016–2021 Fire map of AVA: Santa Cruz Mts; 2016–2021 Fire map of AVAs: Alexander Valley, Sonoma Valley, Sonoma, and Dry Creek; average percentage of PDs in 24 Zinfandel wine samples categorized by smoke impact levels; and Zinfandel composition across all wine samples (PDF)

## Abbreviations

CH_3_CN: acetonitrile
ALX: Alexander Valley
AM: Amador County
AVAs: American Viticulture areas
AWRI: Australian Wine Research Institute
CID: collision-induced dissociation
DRY: Dry Creek
ED or ELDO: El Dorado
ESI: electrospray ionization
ISTD: Internal Standard
IPA: isopropanol
LC–MS: liquid chromatography–mass spectrometry
LDI: Lodi
MeOH: methanol
MRM: multiple reaction monitoring
PSO: Paso Robles
PDs: phenolic diglycosides
PGs: phenolic glycosides
*t*_R_: retention times
RMA: Risk Management Agency
SCC: Santa Cruz Campaign
SCM: Santa Cruz Mountains
SE: sensory evaluation
SPE: solid phase extraction
NaOH: sodium hydroxide
SON: Sonoma County
SNV: Sonoma Valley
UHPLC: ultra-high-performance liquid chromatography
UCCE: UC Cooperative Extension
VZs: viticulture zones

## References

[ref1] Oberholster A.; Wen Y.; Dominguez Suarez S.; Erdmann J.; Cauduro Girardello R.; Rumbaugh A.; Neupane B.; Brenneman C.; Cantu A.; Heymann H. Investigation of Different Winemaking Protocols to Mitigate Smoke Taint Character in Wine. Molecules 2022, 27, 1732 10.3390/molecules27051732. 35268834 PMC8911878

[ref2] Coulter A.; Baldock G.; Parker M.; Hayasaka Y.; Francis I. L.; Herderich M. Concentration of smoke marker compounds in non-smoke-exposed grapes and wine in Australia. Aust. J. Grape Wine Res. 2022, 28, 459–474. 10.1111/ajgw.12543.

[ref3] Summerson V.; Gonzalez Viejo G.; Pang A.; Torrico D. D.; Fuentes S. Review of the effects of grapevine smoke exposure and technologies to assess smoke contamination and taint in grapes and wine. Beverages 2021, 7, 7 10.3390/beverages7010007.

[ref4] Parker M.; Osidacz P.; Baldock G. A.; Hayasaka Y.; Black C. A.; Pardon K. H.; Jeffery D. W.; Geue J. P.; Herderich M. J.; Francis I. L. Contribution of several volatile phenols and their glycoconjugates to smoke-related sensory properties of red wine. J. Agric. Food Chem. 2012, 60, 2629–2637. 10.1021/jf2040548. 22324544

[ref5] Krstic M. P.; Johnson D. L.; Herderich M. J. Review of smoke taint in wine: smoke-derived volatile phenols and their glycosidic metabolites in grapes and vines as biomarkers for smoke exposure and their role in the sensory perception of smoke taint. Aust. J. Grape Wine Res. 2015, 21, 537–553. 10.1111/ajgw.12183.

[ref6] Kropp J. D.; De Andrade M. A. Wildfires and Smoke Exposure Create Contracting and Crop Insurance Challenges for California’s Wine Industry. Choices Mag. 2022, 37, 1–12.

[ref7] Wallace B. When Smoke Gets in Your Wine. 2022, New York Magazine. https://nymag.com/intelligencer/article/california-wildfires-wine-napa-cab.html?regwall-newsletter-signup=true (accessed June 23, 2022).

[ref8] 2021 California Winegrape Crushed 3,613,009 Tons, 2022, Advisor: Wine Industry Network. https://wineindustryadvisor.com/2022/02/11/2021-california-winegrape-crushed-3613009-tons (accessed June 23, 2022).

[ref9] Crews P.; Dorenbach P.; Amberchan G.; Keiffer R. F.; Lizama-Chamu I.; Ruthenburg T. C.; McCauley E. P.; McGourty G. Natural Product Phenolic Diglycosides Created from Wildfires, Defining Their Impact on California and Oregon Grapes and Wines. J. Nat. Prod. 2022, 85, 547–561. 10.1021/acs.jnatprod.2c00028. 35239347 PMC8961875

[ref10] Høj P.; Pretorius I.; Blair R. J. The Australian Wine Research Institute Annual Report; The Australian Wine Research Institute: Urrbrae, SA, Australia, 2003 https://www.awri.com.au/wp-content/uploads/2003_AWRI_Annual_Report.pdf (accessed Dec 2021).

[ref11] Hayasaka Y.; Parker M.; Baldock G. A.; Pardon K. H.; Black C. A.; Jeffery D. W.; Herderich M. J. Assessing the impact of smoke exposure in grapes: development and validation of a HPLC-MS/MS method for the quantitative analysis of smoke-derived phenolic glycosides in grapes and wine. J. Agric. Food Chem. 2013, 61, 25–33. 10.1021/jf305025j. 23230971

[ref12] Dungey K. A.; Hayasaka Y.; Wilkinson K. L. Quantitative analysis of glycoconjugate precursors of guaiacol in smoke-affected grapes using liquid chromatography-tandem mass spectrometry based stable isotope dilution analysis. Food Chem. 2011, 126, 801–806. 10.1016/j.foodchem.2010.11.094.

[ref13] Wilkinson K. L.; Pinchbeck K. A.; Ristic R.; Baldock G. A.; Hayasaka Y. Assessing Smoke Taint in Grapes and Wine. Flavor Chemistry of Wine and Other Alcoholic Beverages; Qian M. S., Shelhammer T. H., Eds.; ACS Symposium Series; American Chemical Society: Washington, D.C., USA, 2012; Vol, 1104, pp 57–65.

[ref14] Modesti M.; Szeto C.; Ristic R.; Jiang W. W.; Culbert J.; Bindon K.; Catelli C.; Mencarelli F.; Tonutti P.; Wilkinson K. Potential mitigation of smoke taint in wines by post-harvest ozone treatment of grapes. Molecules 2021, 26, 1798 10.3390/molecules26061798. 33806831 PMC8004780

[ref15] Caffrey A.; Lerno L.; Rumbaugh A.; Girardello R.; Zweigenbaum J.; Oberholster A.; Ebeler S. E. Changes in smoke-taint volatile-phenol glycosides in wildfire smoke-exposed Cabernet Sauvignon grapes throughout winemaking. Am. J. Enol. Vitic. 2019, 70, 373–381. 10.5344/ajev.2019.19001.

[ref16] Noestheden M.; Dennis E. G.; Romero-Montalvo E.; DiLabio G. A.; Zandberg W. F. Detailed characterization of glycosylated sensory-active volatile phenols in smoke-exposed grapes and wine. Food Chem. 2018, 259, 147–156. 10.1016/j.foodchem.2018.03.097. 29680037

[ref17] Szeto C.; Ristic R.; Capone D.; Puglisi C.; Pagay V.; Culbert J.; Jiang W.; Herderich M.; Tuke J.; Wilkinson K. Uptake and Glycosylation of Smoke-Derived Volatile Phenols by Cabernet Sauvignon Grapes and Their Subsequent Fate during Winemaking. Molecules 2020, 25, 3720 10.3390/molecules25163720. PMC7464031 32824099

[ref18] Fryer J. A.; Collins T. S.; Tomasino E. Evaluation of Different Interstimulus Rinse Protocols on Smoke Attribute Perception in Wildfire-Affected Wines. Molecules 2021, 26, 5444 10.3390/molecules26185444. 34576915 PMC8470714

[ref19] Jiang W. W.; Parker M.; Hayasaka Y.; Simos C.; Herderich M. Compositional Changes in Grapes and Leaves as a Consequence of Smoke Exposure of Vineyards from Multiple Bushfires across a Ripening Season. Molecules 2021, 26, 3187 10.3390/molecules26113187. 34073537 PMC8197810

[ref20] Härtl K.; Huang F.; Giri A. P.; Franz-Oberdorf K.; Frotscher J.; Shao Y.; Hoffmann T.; Schwab W. Glucosylation of smoke-derived volatiles in grapevine (Vitis vinifera) is catalyzed by a promiscuous resveratrol/guaiacol glucosyltransferase. J. Agric. Food Chem. 2017, 65, 5681–5689. 10.1021/acs.jafc.7b01886. 28656763

[ref21] McGourty G. Wine Business Monthly, 2020; Vol. 27, pp 176–183.

[ref22] The Impact of Wildfires, ETS Harvest Guide 2022 Section (pg, 33–38). https://issuu.com/etslabs/docs/harvestquarterly2021digital (accessed August 22, 2022)

[ref23] Nealon S. Oregon State Researchers Discover Compounds Contributing to Smoke Taint in Wine and Grapes, Oregon State University Newsroom. 2002, https://today.oregonstate.edu/news/oregon-state-researchers-discover-compounds-contributing-smoke-taint-wine-and-grapes (acces June 26, 2022) .

