# Supplementary material for: Appraising California Zinfandel Exposure to Wildfire Smoke Using Natural Product Phenolic Diglycoside Biomarkers
Source: J Agric Food Chem. 2022 Sep 8;70(37):11738–48. doi: 10.1021/acs.jafc.2c04807 (PMC9501791; doi:10.1021/acs.jafc.2c04807)
Supplement: Supplementary file 1 — jf2c04807_si_001.pdf [file jf2c04807_si_001.pdf]

Supplementary information

Appraising California Zinfandel Exposure to Wildfire Smoke Using Natural Product Phenolic Diglycoside Biomarkers

Phillip Crews,*† Paul Dorenbach,‡ Gabriella Amberchan,†

†Department of Chemistry and Biochemistry, University of California, Santa Cruz, California 95064, USA.

‡SC Laboratories Inc, Santa Cruz, California 95060, USA.

***Corresponding Author:** Phillip Crews, PhD
Tel.: +1 831 459 2603; fax: +1 831 459 2935
E-mail address: pcrews@ucsc.edu

Table of Contents

	Page
**Table S1.** Total number of fires between 2016 – 2021 in the five selected CA Zinfandel viticulture zones (and the 9 specific AVAs).	3
**Table S2.** 2016 – 2021 Fire map of AVA: Amador.	3
**Table S3.** 2016 – 2021 Fire map of AVA: El Dorado.	4
**Table S4.** 2016 – 2021 Fire map of AVA: Lodi.	5
**Table S5.** 2016 – 2021 Fire map of AVA: Paso Robles.	6
**Table S6.** 2016 – 2021 Fire map of AVA: Santa Cruz Mts.	8
**Table S7.** 2016 – 2021 Fire map of AVAs: Alexander Valley, Sonoma Valley, Sonoma, and Dry Creek.	9
**Figure S1.** Average percentage of PDs in 24 Zinfandel wine samples categorized by smoke impact levels.	10
**Figure S2.** Zinfandel composition across all wine samples	11

Table S1. Total number of fires between 2016 – 2021 in the five selected CA Zinfandel viticulture zones (and the 9 specific AVAs). Data collected from the Cal Fire website (www.fire.ca.gov)

Acreage	Code	Number of Fires	Sierras (Amador)	Sierras (El Dorado)	Lodi (Lodi)	Central Coast (Paso Robles)	Bay Area (Santa Cruz Mtn)	Sonoma (Alexander Valley, Sonoma Valley, Sonoma, & Dry Creek)
>100,000 acres	Extreme Fire	2	0	1	0	0	0	1
1,000 - 99,999 acres	Major Fire	23	0	2	1	11	1	8
500 - 999 acres	Large Fire	5	1	0	0	4	0	0
100 - 499 acres	Medium Fire	40	2	7	4	21	2	4
0 - 99 acres	Small Fire	84	10	25	4	33	7	5
	Total Fires	154	13	35	9	69	10	18

Table S2. Between 2016 – 2021 there were 13 fires in the Amador AVA. Color code: Extreme Fire (>100,000 acres, red), Major Fire (1,000 – 99,999 acres, orange), Large Fire (500 – 1,000 acres, yellow), Medium Fire (100 – 499 acres, blue), Small Fire (0 – 99 acres, green). Data collected from the Cal Fire website (www.fire.ca.gov)

Year	AVA: Amador (Date of Active Fire)	Acres Burned	Lat/Long
2016	Rancho Fire (June 28-29)	372	[38.385, -121.003611]
	Bell Fire (Sept 23 - 25)	45	[38.5212, -120.8416]
	Camanche Fire (June 17- 21)	210	[38.26874, -120.90265]
	Deer Fire (Aug 5 - 6)	26	[38.2581, -120.8657]
	Willow Fire (Aug 30)	19	Off Highway 124 & Willow Creek Rd, 4 miles east of Ione
2017	Sandra Fire (Aug 28)	10	[38.34421, -120.72274]
2018	Irish Fire (July 6)	825	[38.42623, -120.95408]
	China Fire (Jun 17)	17	[38.265645, -120.892404]
2019	Electra Fire (Sept 25)	10	[38.332083, -120.67131]
2020	Lambert Fire (Oct 4)	21	[38.424016, -121.006635]
	Copper (Sept 30)	48	[38.49375, -121.003602]
2021	Lawrence Fire (Sept 5)	46	[38.529123, -120.731748]
	Goose Fire (June 13)	67	[38.3046414, -120.9608439]

Table S3. Between 2016 – 2021 there were 35 fires in the El Dorado AVA. Color code: Extreme Fire (>100,000 acres, red), Major Fire (1,000 – 99,999 acres, orange), Large Fire (500 – 1,000 acres, yellow), Medium Fire (100 – 499 acres, blue), Small Fire (0 – 99 acres, green). Data collected from the Cal Fire website (www.fire.ca.gov)

Year	AVA: El Dorado (Date of Active Fire)	Acres Burned	Lat/Long
2016	Emerald Fire (Oct 13)	176	[38.93449, -120.10744]
	Alder Fire (Aug 6)	25	off of Hwy 50 at Riverton, west of Kyburz
	Storksbill Fire (July 22)	50	[38.79549, -121.00299]
	Aukum Fire (June 3)	25	[38.64107, -120.6955]
	Trailhead Fire (June 28)	5,646	[38.96741, -120.9375]
2017	Holiday Fire (Dec 28)	80	[38.869036, -119.61865]
	Table Fire (Oct 13)	426	[38.848, -120.287]
	Ice Fire (Oct 10)	29	[38.77921, -120.46234]
	French Fire (Sept 17)	136	[38.5758, -120.915]
	Largo Fire (Sept 29)	236	[38.538332, -121.039267]
	Ranch Fire (July26)	140	[38.559707, -120.70013]
	Woodland Fire (July 25)	13	[38.69775, -120.73757]
	Rosado Fire (July 12)	13	[38.67474, -121.06088]
	Axel Fire (July 8)	74	[38.7803, -120.97111]
2018	Meyers Fire (Sept 20)	12	[38.7448, -120.65672]
	Sliger Fire (Sept 4)	150	[38.94388, -120.92611]
	Omega Fire (Aug 1)	66	[38.82128, -121.03918]
	Bumper Fire (Aug 1)	67	[38.645, -120.874167]
	Shingle Fire (July 4)	316	[38.539806, -121.059979]
2019	Caples Fire (Oct 11)	3,435	[38.724, -120.145]
	Chaparral Fire (Sept 29)	22	[38.538467, -120.965105]
	Country Fire (Sept 3)	85	[38.898915, -120.946657]
	Patterson Fire (Aug 15)	35	[38.652475, -120.826165]
	Greenstone Fire (Aug 14)	16	[38.703889, -120.890556]
	Carson Fire (July 30)	13	[38.757636, -120.75664]
2020	Cameron Fire (Oct 18)	15	[38.659387, -120.964726]
	Murphy Fire (Aug 17)	14	[38.840068, -120.876546]
	Sophia Fire (Aug 2)	36	[38.686478, -121.103299]
	Brandon Fire (Jul 7)	32	[38.58488, -120.941267]
	Cronan Fire (Jun 9)	29	[38.827755, -120.988571]
2021	Steins Fire (Jun 26)	11	[38.659971, -120.876096]
	Equestrian Fire (Jun 16)	10	[38.79988, -121.01273]
	Settlers Fire (May 19)	45	[38.56101, -121.01365]
	Salmon Fire (May 1)	32	[38.773294, -121.034998]
	Caldor Fire (Aug 14)	221,835	[38.586, -120.537833]

Table S4. Between 2016 – 2021 there were 9 fires in the Lodi AVA. Color code: Extreme Fire (>100,000 acres, red), Major Fire (1,000 – 99,999 acres, orange), Large Fire (500 – 1,000 acres, yellow), Medium Fire (100 – 499 acres, blue), Small Fire (0 – 99 acres, green). Data collected from the Cal Fire website (www.fire.ca.gov)

Year	AVA: Lodi (Date of Active Fire)	Acres Burned	Lat/Long
2016	Bird Fire (Aug 20)	147	[37.62215, -121.36665]
2018	Waverly Fire (June 29)	12,300	[38.052055, -120.945482]
	Liberty Fire (June 11)	62	[38.24033, -121.09199]
2019	Flood Fire (Sept 5)	44	[38.017138, -120.945044]
	Whiskey Fire (July 16)	90	[37.962557, -121.468074]
	Hollow Fire (July 3-5)	283	[37.63206, -121.538382]
2020	Patterson Fire (July 6)	150	[37.71984, -121.53524]
	Liberty Fire (June 17 -18)	89	[38.23432, -121.20828]
2021	Hollow Fire (Oct. 24)	150	[37.62879, -121.52692]

Table S5. Between 2016 – 2021 there were 69 fires in the Paso Robles AVA. Color code: Extreme Fire (>100,000 acres, red), Major Fire (1,000 – 99,999 acres, orange), Large Fire (500 – 1,000 acres, yellow), Medium Fire (100 – 499 acres, blue), Small Fire (0 – 99 acres, green). Data collected from the Cal Fire website (www.fire.ca.gov)

Year	Paso Robles (Date of Active Fire)	Acres Burned	Lat/Long
2016	Chimney Fire (Aug 13-Sept 6)	46,344	[35.70595, -120.98316]
	Frazier Fire (June 27-29)	86	[35.70821, -120.82266]
	Soda Fire (June 4-19)	0	[35.01382, -119.58206]
	Roberts Fire (May 18-20)	3,712	10 miles northwest of Paso Robles on Camp Roberts Military Base
	Shedd Fire (May 10)	150	[35.61961, -120.39993]
2017	Soda Fire (Oct 20)	21	[35.41112, -120.00353]
	Lynch Fire (Oct 13)	73	[35.78616, -120.96514]
	Pozo Fire (Oct 9)	45	[35.28738, -120.44448]
	Linne Fire (Sept 26)	74	[35.5956, -120.56068]
	Poly Fire (Sept 26)	105	[35.3024, -120.65279]
	Cove Fire (Sept 23)	25	[35.76343, -121.04445]
	Huasna Fire (Sept 20)	245	[35.12269, -120.39387]
	Margarita Fire (Sept 3)	120	[35.31886, -120.50886]
	Yankee Fire (Aug 11)	775	[35.7908, -120.77485]
	Camatta Fire (Aug 2)	70	[35.4403, -120.2913]
	Red Fire (Aug 2)	460	[35.40357, -120.28037]
	Olive Fire (Jul 24)	150	[35.7751, -120.23656]
	Zenon Fire (Jul 20)	47	[35.06472, -120.5634]
	Stone Fire (Jul 9)	340	[35.42433, -120.47322]
	Tower Fire (Jul 7)	58	[35.346, -120.62825]
	Alamo Fire (Jul 9)	28,687	[35.0179, -120.3223]
	Hill Fire (Jun 26)	1,598	[35.4025, -120.4992]
	Lucy Fire (Jun 14)	150	[35.6871, -120.3187]
	Rocky Fire (May 20)	17	[35.556667, -120.474]
	Range Fire (May 19)	0	[35.85253, -120.80411]
	Camp Fire (May 18)	225	[35.34541, -120.76464]
	Arrow Fire (May 15)	225	[35.21177, -119.87525]
	Shandon Fire (May 7)	12	[35.6879, -120.3317]
	Olive Fire (May 2)	130	[35.78286, -120.22647]
2018	Camino Fire (Oct 19)	58	[35.17693, -120.48861]
	Shandon Fire (Sept 29)	80	Gillis Canyon Road and San Juan Road, east of Paso Robles
	Reservoir Fire (Sept 16)	51	[35.30151, -120.64398]
	Rock Fire (Jul 21)	30	[35.68539, -120.95157]

	Mustang Fire (Jul 21)	37	[35.64917, -120.71907]
	Camp Fire (Jul 17)	14	[35.70086, -120.97339]
	Fox Fire (Jun 26)	260	[35.33916, -120.71488]
	Creek Fire (Jun 21)	10	[35.05747, -120.39295]
	Yankee Fire (Jun 20)	1,500	[35.73629, -120.75593]
	Creston Fire (Jun 14)	60	[35.58354, -120.624135]
	Vista Fire (Jun 13)	60	[35.663607, -120.670311]
	Resort Fire (Jun 3)	12	[35.75737, -120.91793]
	Vineyard Fire (May 29)	25	[35.71859, -120.64028]
	Water Fire (May 24)	100	[35.56922, -120.12441]
2019	Bitter Fire (Oct 11)	30	[35.709176, -120.312856]
	Ranch Fire (Oct 1)	52	[35.189783, -120.1755]
	Lopez Fire (Sept 21-27)	220	[35.214364, -120.455883]
	Thompson Fire (Jul 24)	95	[35.004722, -120.435278]
	Gillis Fire (Jul 8-9)	974	[35.63111111, -120.26916667]
	McMillan Fire (Jun 12-24)	1,764	[35.66318, -120.41128]
	Bitter Fire (Jun 10-11)	120	[35.57376, -120.11738]
	Boulder Fire (Jun 5-11)	1,127	[35.343761, -119.913717]
	Belmont Fire (May 29-Jun 3)	835	[35.30759, -119.96498]
2020	Placer Fire (Aug 25)	53	[35.4335197, -120.1243296]
	3-2 Fire (Aug 16-17)	20	[35.519723, -120.801074]
	Whale Fire (Aug 15-19)	312	[35.472114, -120.856731]
	Carrizo Fire (Aug 12)	183	[35.308259, -119.987565]
	Pass Fire (Aug 4-5)	280	[35.61521, -120.40457]
	Pond Fire (Aug 1-9)	1,962	[35.43128, -120.47346]
	Branch Fire (Jul 28-Aug 1)	3,022	[35.35146, -120.00521]
	Lake Fire (Jul 5)	588	[35.351065, -120.00485]
	Soda Fire (Jul 3-5)	157	[35.393889, -120.053275]
	Gage Fire (Jun 28)	33	[35.69993, -120.96759]
	Bend Fire (Jun 25-28)	263	[35.30742, -119.88048]
	River Fire (Jun 22-23)	15	[35.62906, -120.68326]
	Riata Fire(Jun 16-17)	18	[35.40953, -120.56823]
	Avila Fire (Jun 15-19)	445	[35.17977, -120.69959]
	Soda Fire (Jun 10-11)	1,672	[35.35146, -120.00521]
	Range Fire (May 27-28)	5,000	[35.34237, -120.70524]
2021	Tobin Fire (Jul 31)	75	[35.65927, -120.560856]

Table S6. Between 2016 – 2021 there were 10 fires in Santa Cruz Mts AVA. Color code: Extreme Fire (>100,000 acres, red), Major Fire (1,000 – 99,999 acres, orange), Large Fire (500 – 1,000 acres, yellow), Medium Fire (100 – 499 acres, blue), Small Fire (0 – 99 acres, green). Data collected from the Cal Fire website (www.fire.ca.gov)

Year	Santa Cruz Mts. (Date of Active Fire)	Acres Burned	Lat/Long
2017	Bear Fire (Oct 16)	391	[37.18356, -122.07012]
2018	Bear Fire (Nov 16)	10	[37.12857, -122.12036]
	Rincon Fire (Nov 3)	17	[37.0125, -122.046]
2019	Deer Fire (Sept 3)	10	[37.178353, -122.07743]
2020	CZU Lightning Complex (Aug 15-Sept 22)	86,509	[37.17162, -122.22275]
2021	Estrada Fire (Oct 15)	148	[37.013464, -121.752106]
	Freedom Fire (Jan 19)	37	[36.98767, -121.83627]
	Panther Ridge Fire (Jan 19)	20	[37.18381, -122.13372]
	Bonny Doone Complex (Jan 18)	20	[37.08931, -122.10509]
	China Grade Fire (Jan 18)	22	[37.17749, -122.1756]

Table S7. Between 2016 – 2021 there were 18 fires across the AVAs of Alexander Valley, Sonoma Valley, Sonoma, and Dry Creek). Color code: Extreme Fire (>100,000 acres, red), Major Fire (1,000 – 99,999 acres, orange), Large Fire (500 – 1,000 acres, yellow), Medium Fire (100 – 499 acres, blue), Small Fire (0 – 99 acres, green). Data collected from the Cal Fire website (www.fire.ca.gov)

Year	Alexander Valley, Sonoma Valley, Sonoma, & Dry Creek (Date of Active Fire)	Acres Burned	Lat/Long
2016	Sawmill Fire (Sept 25)	1,547	[38.80017, -122.82895]
2017	Oakmont Fire (Central LNU Complex) (Oct 14)	100	[38.46123, -122.58152]
	37 Fire (Oct 9)	1,660	[38.14242, -122.47301]
	Pocket Fire (Central LNU Complex) (Oct 8)	17,357	[38.76549, -122.90939]
	Adobe Fire (Central LNU Complex)	1,868	near Kenwood
	Fort Fire (Oct 7)	20	[38.51937, -123.24713]
	Bodega Fire (Aug 27)	18	[38.26294, -122.71998]
	Independence Fire (July 31)	11	[38.70289, -122.90217]
2018	Pallet Fire (Jun 5)	10	[38.245151, -122.44408]
2019	Kincade Fire (Oct 23)	77,758	[38.792458, -122.780053]
	Tubbs Fire (Central LNU Complex) (Oct 8)	36,807	[38.60895, -122.62879]
	Nuns/Adobe/Norrbom/Pressley/Patrick Fires/ Oakmont (Central LNU Complex) (Oct 8)	56,556	[38.4041, -122.5209]
2020	Glass Fire (Sept 26)	67,484	[38.56295, -122.49745]
	LNU Complex (Aug 16)	363,220	[38.48193, -122.14864]
	Gulch Fire (Jun 6)	107	[38.216134, -122.536406]
	Lakeville Fire (Jun 5)	141	[38.224849, -122.549816]
2021	Fremont Fire (Sept 22)	116	[38.269863, -122.416983]
	Middle Fire (Aug 12)	22	[38.250596, -122.727542]

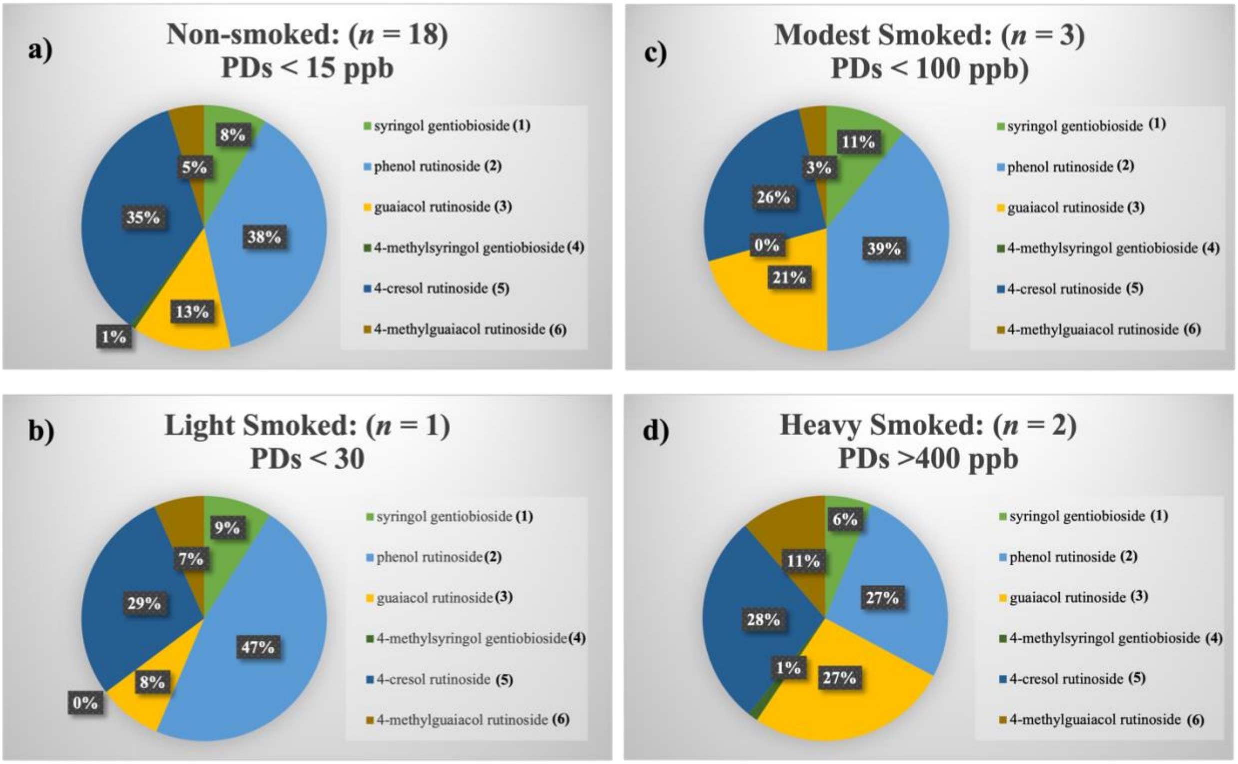

Figure S1. Average percentage of PDs **1 – 6** in the 24 Zinfandel wine samples vs categories of smoke intensity.

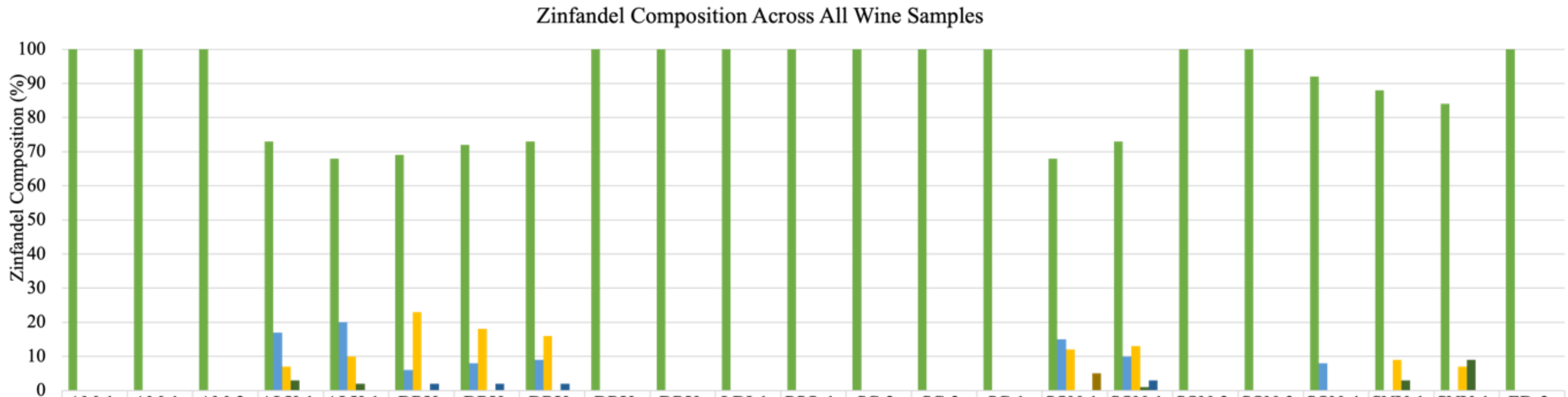

	AM-1-2018 *	AM-1-2021 *	AM-2-2018	ALX-1-2016	ALX-1-2018	DRY-1-2016	DRY-1-2018	DRY-1-2019	DRY-2-2019	DRY-3-2018	LDI-1-2020	PSO-1-2020	SC-2-2016	SC-2-2018	SC-1-2020	SON-1-2016	SON-1-2019 *	SON-2-2019 *	SON-3-2020 *	SON-4-2018	SNV-1-2016	SNV-1-2018	ED-2-2021 *
Zin (%)	100	100	100	73	68	69	72	73	100	100	100	100	100	100	100	68	73	100	100	92	88	84	100
CAR (%)				17	20	6	8	9								15	10			8			
PS (%)				7	10	23	18	16								12	13				9	7	
AB (%)				3	2												1				3	9	
MVD						2	2	2									3						
GRN																5							

Figure S2. The selected Zinfandel wine samples used in this study were composed of varying percentages of the following grape types: Zinfandel (ZIN), Carignan (CAR), Petite Sirah (PS), Alicante Bouschet (AB), and Mourvedre (MVD), and Grenache (GRN). *Data from ref 8.
